# FATHER–CHILD INTERACTIONS AT 3 MONTHS AND 24 MONTHS: CONTRIBUTIONS TO CHILDREN'S COGNITIVE DEVELOPMENT AT 24 MONTHS

**DOI:** 10.1002/imhj.21642

**Published:** 2017-04-27

**Authors:** Vaheshta Sethna, Emily Perry, Jill Domoney, Jane Iles, Lamprini Psychogiou, Natasha E.L. Rowbotham, Alan Stein, Lynne Murray, Paul G. Ramchandani

**Affiliations:** ^1^ King's College London; ^2^ Imperial College London; ^3^ University of Exeter; ^4^ University of Oxford; ^5^ University of Reading and Stellenbosch University; ^6^ Imperial College London

**Keywords:** father–child interactions, cognitive development, early parenting, child development, father–infant interactions, interacciones papá‐niño, desarrollo cognitivo, crianza temprana, desarrollo del niño, interacciones papá‐infante, interactions père‐enfant, développement cognitif, parentage précoce, développement de l'enfant, interactions père‐bébé, Väter‐Kind‐Interaktionen, kognitive Entwicklung, frühe Elternschaft, Kinderentwicklung, Vater‐Säugling‐Interaktionen, 父親と子どもの相互交流、認知 発達、早期の育児、子どもの発達, 父子互動, 認知發育, 早期育兒, 兒童發育, 父子互動

## Abstract

The quality of father–child interactions has become a focus of increasing research in the field of child development. We examined the potential contribution of father–child interactions at both 3 months and 24 months to children's cognitive development at 24 months. Observational measures of father–child interactions at 3 and 24 months were used to assess the quality of fathers’ parenting (*n* = 192). At 24 months, the Mental Developmental Index (MDI) of the Bayley Scales of Infant Development, Second Edition (N. Bayley, 1993) measured cognitive functioning. The association between interactions and cognitive development was examined using multiple linear regression analyses, adjusting for paternal age, education and depression, infant age, and maternal sensitivity. Children whose fathers displayed more withdrawn and depressive behaviors in father–infant interactions at 3 months scored lower on the MDI at 24 months. At 24 months, children whose fathers were more engaged and sensitive as well as those whose fathers were less controlling in their interactions scored higher on the MDI. These findings were independent of the effects of maternal sensitivity. Results indicate that father–child interactions, even from a very young age (i.e., 3 months) may influence children's cognitive development. They highlight the potential significance of interventions to promote positive parenting by fathers and policies that encourage fathers to spend more time with their young children.

## RESUMEN

La calidad de las interacciones papá‐niño se ha convertido en el enfoque de una creciente investigación en el campo del desarrollo del niño. Examinamos la potencial contribución de las interacciones papá‐niño a los 3 y a los 24 meses al desarrollo cognitivo de los niños a los 24 meses. Se usaron las medidas de observación de las interacciones papá‐niño a los 3 y a los 24 meses para evaluar la calidad de la crianza en el caso de los papás (n = 192). A los 24 meses, el Índice de Desarrollo Mental (MDI) de las Escalas de Bailey del Desarrollo del Infante midió el funcionamiento cognitivo. La asociación entre las interacciones y el desarrollo cognitivo se examinó usando análisis de regresión lineal múltiples, con ajustes por la edad de los padres, educación y depresión, edad del infante y sensibilidad materna. Los niños cuyos papás presentaban conductas más despegadas y depresivas en las interacciones papá‐infante a los 3 meses, obtuvieron un puntaje más bajo en MDI a los 24 meses. A los 24 meses, los niños cuyos papás estaban más involucrados y eran más sensibles, y aquellos cuyos papás eran menos controladores en sus interacciones, obtuvieron más altos puntajes en MDI. Estos resultados se dieron independientemente de los efectos de la sensibilidad materna. Los resultados indican que las interacciones papá‐niño, aun a edad temprana (v.g. 3 meses) pudieran influir el desarrollo cognitivo de los niños. Ellos subrayan el significativo potencial de las intervenciones para promover una crianza positiva en los papás, y políticas que animen a los papás a pasar más tiempo con sus niños pequeños.

## RÉSUMÉ

La qualité des interactions père‐enfant est devenue la cible de plus en plus de recherches dans le domaine du développement de l'enfant. Nous avons examiné la contribution potentielle des interactions père‐enfant à 3 mois et à 24 mois au développement cognitif des enfants à 24 mois. Des mesures d'observation des interactions père‐enfant à 3 mois et à 24 mois ont été utilisées pour évaluer la qualité du parentage des pères (n = 192). A 24 mois l'Index de Développement Mental (MDI en anglais) des échelles Bayley du Développement du nourrisson a mesuré le fonctionnement cognitif. Le lien entre les interactions et le développement cognitif a été examiné en utilisant des analyses de régression linéaires multiples, en ajustant l’âge paternel, l’éducation et la dépression paternelles, l’âge du bébé et la sensibilité maternelle. Les enfants dont les pères faisaient preuve de plus de comportements retirés et dépressifs durant les interactions père‐enfants à trois mois avaient des scores plus bas au MDI à 24 mois. A 24 mois les enfants dont les pères étaient moins engagés et sensibles et ceux dont les pères étaient moins autoritaires dans leurs interactions ont reçu des scores plus élevés au MDI. Ces résultats n'ont aucun rapport avec les effets de la sensibilité maternelle. Les résultats indiquent que les interactions père‐enfant, même à un très jeune âge (c'est‐à‐dire à trois mois) peuvent influencer le développement cognitif des enfants. Ils mettent en évidence la possible signification des interventions qui promeuvent le parentage par les pères, et des règles d'intervention qui encouragent les pères à passer plus de temps avec leurs jeunes enfants.

## ZUSAMMENFASSUNG

Die Qualität der Vater‐Kind‐Interaktionen ist zunehmend in den Fokus der Forschung auf dem Gebiet der Kinderentwicklung geraten. Wir untersuchten im Kindesalter von 3 und 24 Monaten den potenziellen Beitrag von Vater‐Kind‐Interaktionen auf die kognitive Entwicklung der Kinder im Alter von 24 Monaten. Beobachtungsverfahren zur Messung von Vater‐Kind‐Interaktionen wurden im Kindesalter von 3 und 24 Monaten verwendet, um die Qualität des väterlichen Erziehungsverhaltens zu beurteilen (n = 192). Nach 24 Monaten wurde mit dem “Mental Developmental Index” (MDI) aus der “Bayley's Scales of Infant Development” die kognitive Entwicklung erfasst. Die Assoziation zwischen Interaktionen und kognitiver Entwicklung wurde unter Verwendung multipler linearer Regressionsanalysen untersucht, und für väterliches Alter, Erziehung und Depression, Säuglingsalter und mütterliche Sensitivität angepasst. Kinder, deren Väter zum Zeitpunkt von 3 Monaten mehr zurückgezogene und depressive Verhaltensweisen in der Vater‐Säuglings‐Interaktion zeigten, erreichten nach 24 Monaten auf dem MDI geringere Werte. Zum Zeitpunkt von 24 Monaten erzielten Kinder, deren Väter in der Interaktion engagierter und sensibler waren, sowie diejenigen Kinder, deren Väter weniger kontrollierend waren, im MDI höhere Werte. Diese Ergebnisse waren unabhängig von den Auswirkungen der mütterlichen Sensitivität. Die Ergebnisse deuten darauf hin, dass Vater‐Kind‐Interaktionen bereits frühzeitig (d.h. mit 3 Monaten) die kognitive Entwicklung des Kindes beeinflussen können. Zudem heben sie die potenzielle Bedeutung von Interventionen hervor, die eine positive Erziehung durch Väter fördern, und eine Politik, die Väter dazu ermutigt, mehr Zeit mit ihren Kleinkindern zu verbringen.

## 抄録

父親と子どもの相互交流の質は、子どもの発達分野で増加する研究の焦点になってきている。私たちは3か月と24か月時の父親と子どもの相互交流の、24か月時の子どもの認知発達への寄与の可能性を検証した。3か月と24か月時の父親と子どもの相互交流の観察による測定が、父親の育児 (n = 192) の質を評価するために使われた。24か月時に、乳児発達のBayley尺度の精神発達指標the Mental Developmental Index (MDI) of the Bayley's Scales of Infant Developmentで認知機能が測定された。相互交流と認知発達の関連は、重回帰分析を用いて、検証された。その際、親の年齢、教育および抑うつ、乳幼児の月齢、そして母親の感受性が調整された。3か月時に父親と乳児の相互交流で、より引きこもって抑うつ的な行動を示した父親の子どもは、24か月時にMDIが低い得点だった。24か月時に、より関わり合って感受性がある父親の子ども、そして相互交流の中でコントロールの少ない父親の子どもは、MDIでより高い得点だった。これらの所見は、母親の感受性の効果とは独立していた。結果から、父親と子どもの相互交流は、非常に幼い月齢 (すなわち、3か月) からでも、子どもの認知発達に影響するだろうと、示唆された。それらは、父親によるポジティブな育児を促進する介入の潜在的な意味と、父親が幼い子どもと共に多くの時間を過ごすことを奨励する政策を強調する。

## 摘要

父子互動的素質已成為兒童發展日益增長的研究重點。我們審查在3個月和24個月時父子互動 對24個月時兒童認知發育的潛在貢獻。我們用3和24個月父子互動的觀察量表來評估父親的養育質量 (n = 192) 。 24個月時, 以Bayley的“嬰兒發展量表”的心理發育指數 (MDI) 衡量認知功能。我們用多元線性回歸, 分析互動和認知發展的關聯, 並在分析中調整父親年齡丶教育和抑鬱症丶嬰兒年齡和母親敏感度。在3個月時父子互動中表現較多退縮和抑鬱行為的父親之孩子, 在24個月時MDI得分較低。較多參與丶較敏感和在互動中較少控制的父親之孩子, 在24個月時MDI得分較高。這些發現與母親敏感性的影響無關。結果表明, 即使非常年輕時的父子互動 (即3個月) , 也可能影響到兒童的認知發展。結果強調促進父親積極養育的干預措施之潛在意義, 及鼓勵父親與子女花更多時間在一起的政策之重要性。

## Abstract (ar)

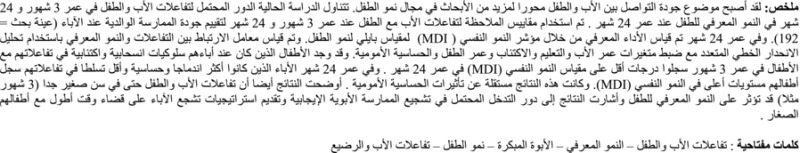

Favorable environmental experiences, especially those embedded within early caregiving relationships, have a positive impact on a child's cognitive development (Bernier, Carlson, & Whipple, 2010). However, while there is compelling support for this association in mothers, less is known about the association between father–infant interactions and children's cognitive development. There has been evidence of positive benefits of fathers’ presence in their children's homes, fathers sensitive parenting (Bornstein, Hahn, & Haynes, 2004; Sarkadi, Kristiansson, Oberklaid, & Bremberg, 2008), and their increased involvement in childcare on other child outcomes such as emotional and behavioral development (Lamb, 2010; Pleck & Masciadrelli, 2004). However, evidence regarding the important domain of cognitive development has been sparse—existing evidence mainly includes older children and disadvantaged samples, and to our knowledge, no study has utilized observational measures of very early father–infant interactions.

A substantial body of evidence has suggested that fathers are critical for child well‐being. For example, while there are positive benefits of father involvement on a range of child outcomes throughout development (Panter‐Brick et al., 2014), available research also has indicated the numerous risks associated with father absence (e.g., Amato, 1994). Furthermore, although mothers continue to contribute the majority of their time to children, paternal involvement in caregiving has increased, especially in middle‐socioeconomic families (Paquette & Bigras, 2010; Yeung, Sandberg, Davis‐Kean, & Hofferth, 2001). Consequently, studies have not only focussed on the quantity of time fathers spend with their children but also (although to a lesser extent) on the quality of their interactions. These observational studies have suggested that even though parents display similarities in their interaction styles, father–child interactions have a distinct quality: more stimulating, vigorous, and arousing in comparison to mother–child interactions (Dixon et al., 1981; MacDonald & Parke, 1986). Their interactive episodes promote their child's risk‐taking and exploration tendencies (Kromelow, Harding, & Touris, 1990), which in turn may facilitate the development of children's cognitive skills. Thus, early social experiences with fathers and mothers may differentially impact children's development (Cabrera, Shannon, & Tamis‐LeMonda, 2007; Hunter, McCarthy, MacTurk, & Vietz, 1987). Therefore, it may be equally important to consider how fathers impact early child development, in addition to studying the influence of mothers.

For instance, a positive association has been reported between paternal involvement in caretaking tasks (as early as the first month of infancy) and Bayley Mental Development Index (MDI; Bayley, 1993) scores 1 year later (Nugent, 1991). In another study, increased father engagement with preterm children (measured via maternal interview) was associated with improved cognitive outcome on the Stanford‐Binet Intelligence Scale at 36 months (Yogman, Kindlon, & Earls, 1995). More recent evidence has suggested that paternal positive affect and cognitive stimulation in play and caregiving (obtained via paternal self‐report) with children aged 9 months benefits their concurrent cognitive skills (Bronte‐Tinkew, Carrano, Horowitz, & Kinukawa, 2008). Yet, other studies utilizing diverse samples and methodologies have failed to find an association (Cabrera, Shannon, West, & Brooks‐Gunn, 2006; Hunter et al., 1987; Magill‐Evans & Harrison, 2001). For instance, there was no association between self‐report measures of paternal involvement at 9 months and cognitive scores at the same time point in Latino infants (Cabrera et al., 2006).

Research on father–child interactions in toddlerhood has provided more consistent results than those on infancy. For instance, supportive paternal behaviors at 2 years were associated with children's intellectual functioning scores at 2 and 3 years of age (Cabrera, Shannon, & Tamis‐LeMonda, 2007). Another study has shown an association between paternal supportiveness at 14 months and increased cognitive ability at 24 and 36 months in low‐income children facing developmental risks (Jeon, Peterson, & DeCoster, 2013). Overall, studies have demonstrated that supportive, sensitive, and stimulating paternal behaviors during toddlerhood are positively associated with children's cognitive outcomes after controlling for various demographic and socioeconomic factors (Cabrera, Fitzgerald, Bradley, & Roggman, 2007; Cabrera, Shannon, & Tamis‐LeMonda, 2007; Shannon, Tamis‐LeMonda, London, & Cabrera, 2002; Tamis‐LeMonda, Shannon, Cabrera, & Lamb, 2004).

Thus far, the vast majority of studies that have examined how fathers might influence their children's cognitive development have largely focused on disadvantage, including father‐absent families (Marsiglio, Amato, Day, & Lamb, 2000), low‐income families (Cook, Roggman, & Boyce, 2011; Jeon et al., 2013; Ryan, Martin, & Brooks‐Gunn, 2006), preterm babies (Magill‐Evans & Harrison, 2001; Yogman et al., 1995), and children with rare medical conditions (McGrath, Wypij, Rappaport, Newburger, & Bellinger, 2004). Consequently, these results may not be applicable to lower risk samples from middle and higher socioeconomic functioning. Not only does the diversity of samples and methodologies used in previous research make it difficult to draw firm conclusions but available studies also were subject to a number of methodological limitations. For example, maternal reports of the parenting strategies of children's fathers are often used, and these may be prone to bias (Mikelson, 2008; Yogman et al., 1995). Even when paternal reports of early involvement are used (Bronte‐Tinkew et al., 2008), they can lack the independence and detail provided by observational methods. Moreover, the majority of studies have measured fathers’ parenting and child development concurrently (Bronte‐Tinkew et al., 2008; Yarrow et al., 1984). Critically, few prospective studies exist that have examined the father's early contribution to later cognitive abilities (Nugent, 1991). Although these prospective studies have fallen short of providing evidence of a causal effect from paternal parenting and later child developmental outcomes, they do provide stronger evidence than do cross‐sectional studies.

Therefore, the current study aims to examine the association between father–infant interactions (at 3 and 24 months) and children's cognitive skills at 24 months. In so doing, we address several gaps in the existing literature: first, to examine the association between father–child interactions (in the 3‐month postnatal period as well as at 24 months) and child cognitive outcomes (measured at 24 months), thereby including interactions at a younger age than have most studies. The early focus of this study is critical, due to the infant's rapid development and high susceptibility to the quality of interactions with parents at this age. Second, this study utilizes observational measures of interactions, allowing for independent examination of a range of early parenting dimensions in different interaction settings. Moreover, at 24 months, in addition to using a free‐play session (i.e., without toys), we also use a joint book session. Although there is evidence from the maternal literature on the implications of a book‐sharing context for cognitive development (e.g., Blake, Macdonald, Bayrami, Agosta, & Milian, 2006), there is much less literature on fathers. The available studies on fathers have largely focused on low‐income families (Pancsofar & Vernon‐Feagans, 2010) and have included the frequency of book‐reading rather than specific paternal behaviors in this interaction context (Duursma, Pan, & Raikes, 2008). Thus, this study aims to understand the cognitive development of children from socioeconomically diverse families, in different interactive settings, whereby the quality of father–child interactions are assessed. Third, an independent, blinded assessment of cognition is utilized. Fourth, and as indicated, the vast majority of evidence focuses on families from lower socioeconomic contexts. Although understandable, due to the possibility that the family's circumstances impact on their ability to develop positive parent–child relationships (Shannon et al., 2002), there has been relatively little evidence of the relationship between father–infant interactions and child cognitive outcomes in families from middle and higher socioeconomic backgrounds. Fifth, we examine the independent effect of fathers on child cognitive development, controlling for paternal depression, age, education, maternal sensitivity, and infant's age. Father's age is related to involvement with his child (Pleck, 1997), and the experience of adverse parenting is associated with parenthood at an early age (Pogarsky, Thornberry, & Lizotte, 2006). Father's educational qualifications are linked to both the study exposure—poorly educated fathers often find it harder to establish positive and sensitive relationships with their children (Cabrera, Shannon, & Tamis‐LeMonda, 2007; Gavin et al., 2002; Tamis‐LeMonda et al., 2004) and outcome—well‐educated parents are particularly sensitive to their infant's development and are more likely to expose their child to cognitive‐stimulating experiences (Cabrera, Shannon, & Tamis‐LeMonda, 2007; Tamis‐LeMonda et al., 2004). Empirical evidence also has suggested that paternal depression is associated with parenting impairment in fathers (for a review, see Wilson & Durbin, 2010) and also may have implications for children's cognitive outcomes (Paulson, Keefe, & Leiferman, 2009). Maternal sensitivity is related in predicted ways to children's cognitive development (e.g., Beckwith & Rodning, 1996; Eshel, Daelmans, Cabral de Mello, & Martines, 2006; Field et al., 1985; Lemelin, Tarabulsy, & Provost, 2006). Moreover, sensitivity comprises aspects of interaction (i.e., warm and sensitive support, responsive and contingent parenting providing appropriate levels of stimulation) that have consistently been associated with improved cognitive ability. In addition to these factors, we also will control for infant's age at the time of assessment because it is likely that an older child more advanced in their social and cognitive development will influence the play session between father and child (Roopnarine, Krishnakumar, Metindogan, & Evans, 2006).

Finally, we examine whether the influence of father–child interactions on cognitive development is moderated by infant gender. Some findings have suggested that fathers influence the development of sons more than they influence daughters (Bronte‐Tinkew et al., 2008; Mott, Kowaleski‐Jones, & Menaghan, 1997). Fathers have been found to be more responsive to boy's affective states (Feldman, 2003), and less sensitive and engaged in play and caretaking activities with their infant daughters, as compared to their sons (Manlove & Vernon‐Feagans, 2002; Schoppe‐Sullivan et al., 2006). As a result of these varying interaction styles, it is possible that fathers may differentially influence sons and daughters in terms of their subsequent cognitive development. However, caution should be exercised in this regard, as not all studies have shown these gender effects (Flouri & Buchanan, 2004; Pougner, Serbin, Stack, & Schwartzman, 2011).

We hypothesize that positive patterns of father–infant interactions at both 3 months and 24 months will contribute to children's cognitive functioning at 24 months, over and above any effects of maternal sensitivity, infant age, and paternal age, education, and psychopathology. Furthermore, we predict that there will be a stronger association between father–child interactions and cognitive functioning for sons than that for daughters.

## METHOD

### Design and Sample

Participants were recruited from the maternity wards of two hospitals in the United Kingdom (Ramchandani et al., 2011). Eligibility criteria included parent's age 18 or over at the time of the child's birth, fluent English, infant's birth weight 2,500 g or more, gestation 37 weeks or more, and no congenital abnormalities. Mothers, fathers, and their infant took part in home assessments when the infant was 3 months and 24 months. Child outcome data, at the 24‐month time point, were collected between 2008 and 2010. Parents gave informed consent. Ethical approval was granted by the Oxfordshire Research Ethics Committee.

### Sample at 3 Months and 24 Months

At the 3‐month assessment, 192 fathers and their infants took part in the study. Fathers had a mean age of 35 years (*SD* = 5.86 years, range = 19–55 years), and the majority were White (94.6%) and either married or living with a partner (99.5%). Infants had a mean age of 14.5 weeks (*SD* = 3.0, range = 10–41 weeks) at this point, and approximately half of the sample (52.6%) were female. Families were contacted again when their children were 24 months old (*M* infant age = 25.15, *SD* = 2.29); 156 (81%) agreed to participate. There was no difference between those who did and did not complete the 24‐month visit in terms of paternal age, *t*(189) = 1.135, *p* = .258, education, χ^2^(3) = 3.09, *p* = .377, and employment status, χ^2^(3) = 0.75, *p* = .862.

### Procedure

**Figure 1:**
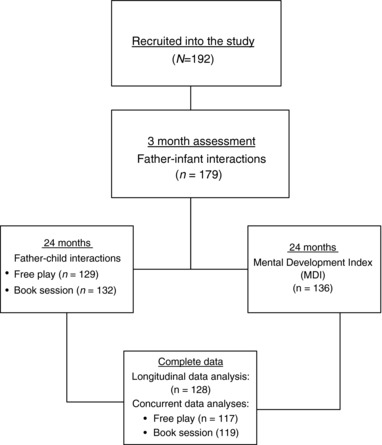
Participant flow through the study stages.

At 3 months, father–child interactions were video‐recorded at home in a floor‐mat setting. Fathers were asked to talk to and play with their infant, for 3 min, as they would normally, without the use of any toys. Similar assessments of parent–infant face‐to‐face interactions have been used in previous studies (Feldman, Greenbaum, & Yirmiya, 1999; Murray, Fiori‐Cowley, Hooper, & Cooper, 1996; Sethna, Murray, Netsi, Psychogiou, & Ramchandani, 2015). Of the total sample (*N* = 192), data were available for 179 participants. Missing data were due to child distress, refusal to be filmed, or technical difficulties. At 24 months, father–child interactions were recorded in two (home‐based) interactive settings: 2 min of “free‐play” without the use of any toys and a 5‐min “book session” with the use of a book. Data were available for 129 fathers in the free‐play session and 132 fathers in the book session. Children were tested at 24 months with the Bayley Scale of Infant Development, Second Edition (BSID‐II; Bayley, 1993). Data were available on 136 children. Complete data on 3‐month father–infant interactions and 2‐year cognitive‐development outcomes were available for 128 participants. Likewise, at the 24‐month time point, complete data were available on 117 participants in the free‐play session and 119 participants in the book session. These samples are used in the present analyses (see Figure 1).

Measures

Father–infant interactions at 3 months.

Interactions were coded using the Global Rating Scales (GRS; Murray et al., 1996). Thirteen paternal behaviors were rated on a series of 5‐point scales (1–5), with lower scores indicating inadequate interactions. Dimensions of paternal interaction derived as per standard use in previous studies include: (a) Sensitivity (paternal response to the infant's communication cues in a way that is appropriate to the infant's needs and experiences, including attitude and feelings toward the infant, Cronbach's α = 0.72); (b) Intrusiveness (overstimulating vocal and physical activity around the infant, cutting across infant communication, Cronbach's α = 0.44); (c) Remoteness (withdrawal and disengagement, Cronbach's α = 0.90); and (d) Depressive Affect (affective state and level of enjoyment, including anxious, vocal, and physical activity, Cronbach's α = 0.57). Twenty percent of interactions were independently coded for interrater agreement. Interrater intraclass correlations (Shrout & Fleiss, 1979) ranged from .74 to .88. Discrepancies between raters were discussed, and final ratings were determined in collaboration with members of the Winnicott Research Unit who were involved in the development of the scale.

#### Father–child interactions at 24 months

Father–child interactions were coded by trained researchers who were not involved in coding the 3‐month interactions. A coding scheme (Madden et al., 2015), adapted from the original domains of the GRS scales, but also including a broader range of behaviors displayed by both the child and father (e.g., physicality in their interactions) at this later phase of development, was used. This is because the GRS is only applicable for coding the interactions of mothers and their children in early infancy. Twenty‐two items were coded for parent behaviors and subsequently subjected to principle component factor analysis.

Three factors (Sensitivity, Control, and Engagement) emerged from the free‐play session, which explained 66% of the variance in the data: Sensitivity had an eigenvalue of 2.96 and explained 32.9% of the variance. This factor was weighted onto by positive expressed emotion, emotional tone, and reciprocity and synchronicity ‐ Cronbach's α = 0.73. Control had an eigenvalue of 1.82 and explained 20.2% of the variance. This factor weighted onto by intrusions and negative expressed emotion, conflictual behavior, and low sensitivity ‐ Cronbach's α = 0.39. Engagement had an eigenvalue of 1.28 and explained 13.1% of the variance. This factor was explained by the father's ability to follow the child's attention and increased engagement and communication ‐ Cronbach's α = 0.59.

Three factors (Sensitivity, Control, and Cognitive Stimulation) emerged from the book session, explaining 52% of the variance in the data. Sensitivity had an eigenvalue of 4.44 and explained 31.7% of the variance. This factor was explained by warmth, reciprocity and synchronicity, positivity in expressed emotion and emotional tone, and following the child's attention and elaborating on their speech, Cronbach's α = 0.77. Control had an eigenvalue of 1.62 and explained 1.6% of the variance. This factor was weighted onto by conflictuous behavior, instrumental touching, strong control, and negative emotion, Cronbach's α = 0.39. Cognitive Stimulation had an eigenvalue of 1.21 and explained 8.6% of the variance. The factor was weighted onto by educational references and displays of positive affect, Cronbach's α = 0.44.

There was moderate to good interrater agreement on each dimension, with the average weighted κ ranging from 0.56 to 0.69.

#### Cognitive development

Children's cognitive functioning was assessed using the BSID‐II (Bayley, 1993), which provided a standardized Mental Development Index (MDI) score. The indexed scale has a mean of 100 and an *SD* of 15. The mean MDI score for our sample was 97.50 (*SD* = 13.35).

#### Study covariates

The Structured Clinical Interview for *Diagnostic and Statistical Manual of Mental Disorders, Fourth Edition* (American Psychiatric Association, 1994) Axis 1 Disorders (SCID First; Spitzer, Gibbon, & Williams, 2002) was used to diagnose major depressive disorder at 3 months and 24 months. Paternal age (years) and education (no qualifications, GCSEs, A levels or equivalent, diploma or equivalent, degree, postgraduate) were assessed at 3 months. Maternal sensitivity was assessed during observed mother–infant interactions by blinded raters at both 3 months using the GRS (Murray et al., 1996) and at 24 months (Madden et al., 2015). Infant age (months) was determined at both study points.

### Statistical Analysis

Statistical analysis was performed using SPSS Version 21.0 (IBM Corp, Armonk, NY), with significance set at *p* < .05. First, we examined correlations between paternal interaction dimensions at 3 months and at 24 months. Second, independent simple linear regression analyses were conducted to test the univariate associations between each of the father–infant interaction dimensions at 3 months and MDI scores. Third, where an association was found, we applied the PROCESS macro tool (Hayes, 2013) to estimate and test the adjusted associations and also to examine whether the interaction between each paternal dimension and gender (Paternal Interaction Dimension × Gender) predicted cognitive development. The following covariates were included in the individual models tested: paternal age, education, and depression; and infant age and maternal sensitivity. PROCESS applies bias‐corrected bootstrapping intervals to probe the interaction term and make inferences about indirect effects rather than relying on the normality assumption. The number of bootstrap samples used to determine 95% bias‐corrected bootstrap confidence intervals was 10,000. ROCESS also produces the conditional effects of the independent variable at the two values of a binary moderator (Gender: male = 0, female = 1). These steps were repeated to examine concurrent associations between father–child interactions and cognitive development at 24 months.

## RESULTS

### Associations Between Paternal Interaction Dimensions at 3 Months and at 24 Months (Table 1)

Fathers who were less remote in their interactions at 3 months (higher scores on the GRS) showed increased positive‐responsiveness, *r* = 0.198, *p* = .028, and engagement, *r* = 0.245, *p* = .006, in the free‐play context at 24 months, and also were more sensitive in the book session, *r* = 0.203, *p* = .022. There was evidence of a weak positive association (at trend level) between paternal remoteness in free‐play at 3 months and cognitive stimulation in the book session at 24 months, *r* = 0.150, *p* = .093; that is, fathers who were less remote at 3 months made more educational references with displays of positive affect in the book session at 24 months. Furthermore, fathers who displayed positive affect (higher scores on the GRS) during the 3‐month interactions also were likely to be more engaged in the free‐play session at 24 months, *r* = 0.230, *p* = .011, and more sensitive in the book session, *r* = 0.165, *p* = .064; – the latter at trend level only.

**Table 1:** Interrelations Between Measures of Father–Child Interactions at 3 Months and 24 Months

Interaction Dimensions	1	2	3	4	5	6	7	8	9	10
3 Months										
1. Sensitivity	1									
2. Intrusiveness	.438^b^	1								
3. Remoteness	.153^a^	−.233^b^	1							
4. Depressive Affect	.279^b^	−.100	.572^b^	1						
24 Months (free‐play)										
5. Sensitivity	−.023	−.047	.198^a^	.082	1					
6. Control	−.077	−.029	.070	−.065	−.150	1				
7. Engagement	−.041	.017	.245^b^	.230^a^	.270^b^	−.077	1			
24 Months (book session)										
8. Sensitivity	.146	.001	.203^a^	.165 (0.064)	.239^b^	−.159	.173	1		
9. Control	−.055	−.088	.119	.083	.042	.137	−.053	−.148	1	
10. Cognitive Stimulation	−.031	.038	.150 (0.093)	.024	.142	.039	.111	.285^b^	−.015	1

a Correlation significant at the .05 level.

b Correlation significant at the .01 level.

### Three‐Month Father–Child Interactions and Cognitive Development

There was evidence of a marginally significant association between paternal sensitivity and cognitive development, β = 0.16, *p* = .086, which was largely unchanged when adjusting for covariates—infants of sensitive fathers had higher MDI scores (seeTable 2). Furthermore, there was no evidence of moderation by infant gender on the association between paternal sensitivity and cognitive development—since the interaction term did not reach significance (see Table 2).

**Table 2:** Independent Models Examining 3‐Month Father–Child Interaction Dimensions and Cognitive Development at 24 Months (– = 128)

3‐Month Interaction Dimensions	Coefficient (*B*)	*p*	(95% CI)	Model Summary
Sensitivity (*M* = 3.71, *SD* = 0.04)				*R* ^2^ = 0.130, *F* = 2.02, *p* = .051
Paternal Sensitivity	3.87	.074	−0.38, 8.12	
Infant Gender	4.97	.039	0.26, 9.69	
Infant Gender × Paternal Sensitivity	2.94	.498	−5.63, 11.51	
Age (Father)	−0.02	.940	−0.41, 0.38	
Education (Father)	1.27	.080	−0.15, 2.68	
Depression (Father)	−1.98	.303	−5.76, 1.81	
Age (Infant)	−0.02	.972	−1.08, 1.04	
Sensitivity (Mother)	3.81	.050	−0.00, 7.63	
Remoteness (*M* = 3.59, *SD* = 0.06)				*R* ^2^ = 0.15, *F* = 2.32, *p* = .025
Paternal Remoteness	3.28	.018	0.57, 5.98	
Infant Gender	4.51	.058	−0.15, 9.17	
Infant Gender × Paternal Remoteness	0.13	.963	−5.17, 5.42	
Age (Father)	0.01	.960	−0.377, 0.40	
Education (Father)	1.37	.056	−0.04, 2.77	
Depression (Father)	−2.09	.271	−5.83, 1.65	
Age (Infant)	0.07	.890	−0.98, 1.13	
Sensitivity (Mother)	3.89	.043	0.13, 7.67	
Depressive Affect (*M* = 4.02, *SD* = 0.04)				*R* ^2^ = 0.15, *F* = 2.43, *p* = .019
Paternal Depressive Affect	5.68	.012	1.27, 10.08	
Infant Gender	4.53	.056	−0.12, 9.17	
Infant Gender × Paternal Depressive Affect	1.54	.725	−7.11, 10.19	
Age (Father)	0.02	.939	−0.37, 0.40	
Education (Father)	1.36	.056	−0.04, 2.76	
Depression (Father)	−2.18	.250	−5.91, 1.55	
Age (Infant)	0.05	.924	−0.99, 1.10	
Sensitivity (Mother)	2.76	.151	−1.02, 6.54	

GRS interaction dimensions scored on a scale from 1–5; low scores indicate poor interactions. Gender: 0 = male, 1 = female. CI = confidence interval.

In contrast, paternal intrusiveness was not associated with child cognitive development, β = 0.017, *p* = .850; hence, no further analyses were conducted on this dimension.

Paternal remoteness was significantly associated with 24‐month MDI scores, β = 0.20, *p* = .035; infants of engaged fathers had higher MDI scores at 24 months. While this association remained significant when adjustments were made for covariates, there was no evidence of moderation by infant gender on the association between paternal remoteness and child cognitive skills (see Table 2).

Similarly, paternal depressive affect was significantly associated with 24‐month MDI scores, β = 0.25, *p* = .008; infants of fathers whose affective state was positive (higher GRS scores) had higher MDI scores at 24 months. This association remained when adjusting for covariates (see Table 2). Furthermore, while there was a marginally significant association between gender and MDI scores, B = 4.53, *p* = .056, implying increased cognitive skills in female infants, the interaction term (Infant Gender × Paternal Depressive Affect) was not significant (see Table 2). Thus, there was no evidence of moderation by infant gender on the association between paternal depressive affect and child cognitive skills.

### Concurrent Associations Between Father–Infant Interactions and Cognitive Development at 24 Months

#### Free‐play session

Increased paternal engagement (i.e., high levels of sensitive, attentive, and involved play with fathers) and decreased Control (i.e., less intrusive and conflictual behaviors) were associated with increased cognitive abilities (i.e., higher MDI scores) at 24 months, β = 0.23, *p* = .024 and β = −0.20, *p* = .046, respectively. Next, when adjusting for covariates, the concurrent associations between paternal engagement and Control during free‐play and children's MDI scores at 24 months were not significant (see Table 3). Sensitivity was not associated with MDI scores, β = 0.06, *p* = .523; hence, no further analyses were conducted on this dimension.

**Table 3:** Independent Models Examining Concurrent Associations Between Father–Child Interaction Dimensions During Free‐Play at 24 Months and Cognitive Development

24‐Month Dimensions (free‐play session)	Coefficient (*B*)	*p*	(95% CI)	Model Summary
Control				
Paternal Control	−2.76	.132	−6.38, 0.85	*R* ^2^ = 0.084, *F* = 1.01, *p* = .433
Infant Gender	1.53	.596	−4.18, 7.24	
Infant Gender × Paternal Control	−1.29	.752	−9.34, 6.77	
Age (Father)	−0.03	.906	−0.54, 0.48	
Education (Father)	1.42	.115	−0.35, 3.18	
Depression (Father)	−1.71	.450	−6.19, 2.77	
Age (Infant)	0.01	.999	−1.16, 1.16	
Sensitivity (Mother)	1.49	.304	−1.37, 4.35	
Engagement				
Paternal Engagement	2.24	.086	−0.10, 5.58	*R* ^2^ = 0.10, *F* = 1.24, *p* = .287
Infant Gender	1.34	.642	−4.39, 7.07	
Infant Gender × Paternal Engagement	3.34	.397	−4.46, 11.13	
Age (Father)	−0.03	.913	−0.53, 0.48	
Education (Father)	1.41	.112	−0.34, 3.17	
Depression (Father)	−1.59	.48	−6.02, 2.84	
Age (Infant)	−0.07	.904	−1.22, 1.08	
Sensitivity (Mother)	1.62	.26	−1.25, 4.50	

Gender: 0 = male, 1 = female. CI = confidence interval.

Furthermore, there was no evidence of moderation by infant gender on any of the associations examined in the free‐play context (Table 3).

#### Book session

The paternal sensitivity, β = 0.31, *p* = .001, control, β =  −0.31, *p* = .002, and cognitive stimulation, β = .25, *p* = .011, dimensions were associated with MDI scores, implying that children obtained higher scores on cognitive functioning when their fathers displayed increased levels of warmth, reciprocity, and positivity, and low levels of control and conflictual behaviors. When adjusting for covariates, these associations remained (Table 4). However, and similar to the free‐play session, there was no evidence of moderation by infant gender on any of the associations examined in the book session (Table 4).

**Table 4:** Independent Models Examining Concurrent Associations Between Father–Child Interaction Dimensions During the Book Session at 24 Months and Cognitive Development

24‐Month Dimensions (book session)	Coefficient (*B*)	*p*	(95% CI)	Model Summary
Sensitivity				*R* ^2^ = 0.18, *F* = 2.48, *p* = .018
Paternal Sensitivity	3.98	.003	1.42, 6.53	
Infant Gender	1.55	.583	−4.05, 7.15	
Infant Gender × Paternal Sensitivity	−1.09	.684	−6.40, 4.22	
Age (Father)	−0.12	.62	−0.58, 0.35	
Education (Father)	1.17	.157	−0.46, 2.80	
Depression (Father)	−2.04	.288	−5.82, 1.75	
Age (Infant)	0.075	.894	−1.04, 1.19	
Sensitivity (Mother)	3.41	.036	0.23, 6.60	
Control				*R* ^2^ = 0.17, *F* = 2.34, *p* = .025
Paternal Control	−4.25	.010	−7.47, −1.03	
Infant Gender	−0.26	.926	−5.85, 5.32	
Infant Gender × Paternal Control	−2.77	.452	−10.06, 4.52	
Age (Father)	−0.07	.751	−0.54, 0.39	
Education (Father)	1.41	.091	−0.23, 3.05	
Depression (Father)	−1.97	.306	−5.76, 1.83	
Age (Infant)	−0.04	.947	−1.16, 1.09	
Sensitivity (Mother)	3.12	.059	−0.13, 6.36	
Cognitive Stimulation				*R* ^2^ = 0.17, *F* = 2.31, *p* = .026
Paternal Cognitive Stimulation	2.57	.037	1.72, 6.87	
Infant Gender	0.28	.92	−5.34, 5.89	
Infant Gender × Paternal Cognitive Stimulation	8.69	.105	−1.86, 19.24	
Age (Father)	−0.01	.957	−0.48, 0.45	
Education (Father)	1.45	.081	−0.18, 3.08	
Depression (Father)	−1.84	.338	−5.64, 1.95	
Age (Infant)	−0.01	.980	−1.13, 1.10	
Sensitivity (Mother)	2.99	.069	−0.24, 6.23	

Gender: 0 = male, 1 = female. CI = confidence interval.

## DISCUSSION

The results of this study indicate that specific dimensions of father–child interactions at both time points are associated with MDI scores even when adjusting for paternal depression, age, and education, and maternal sensitivity and infant age. These dimensions include paternal remoteness and depressive affect at 3 months; engagement during free‐play at 24 months; and sensitivity, cognitive stimulation, and control during the book session at 24 months. There was no robust evidence found of differential effects on boys or on girls. To our knowledge, this is the first longitudinal investigation to study the how father–child interactions as early as 3 months of age influence children's cognitive development. Thus, knowing that the association between interactions and cognitive outcome is evident at a very early age highlights the importance of putting preventive measures in place in early infancy to support fathers to better interact with their children.

### Three‐Month Father–Child Interactions and Cognitive Development

Children whose fathers demonstrated increased remoteness and depressive affect in their interactions obtained lower scores on the MDI. These findings are consistent with previous evidence which has found that highly involved fathers promote a higher level of cognitive competence in their children (Bronte‐Tinkew et al., 2008), and can be explained in a number of ways. It is likely that remote fathers use fewer verbal and nonverbal strategies to communicate with their infants, thereby reducing the infant's social learning experience. Moreover, the first year of life is a period characterized by rapid advances in language and other symbolic competencies (Lamb, 1997). More withdrawn fathers also may provide a less stimulating social environment, which may thus impact the child's cognitive skills. Alternatively, the link between paternal remoteness and depressive affect with child MDI scores could be explained by paternal cognitive skills that are inherited by the child. Since a genetic component for cognitive functioning has been described (Jester et al., 2009; Polderman et al., 2007), we cannot rule out that the association between paternal behaviors and child cognitive skills is a result of the genetic inheritance of cognitive skills from parent to child. In line with this explanation, it also is possible that children may model cognitive styles and affective responses of the father, which in turn may influence paternal interactions and subsequent cognitive skills (Kane & Garber, 2004).

### Father–Child Interactions and Cognitive Development at 24 Months

During free‐play, increased paternal engagement (i.e., involved and attentive paternal behaviors) was associated with higher MDI scores. This finding also is consistent with previous research, indicating that highly engaged fathers are more likely to promote positive cognitive outcomes in toddlerhood (Conner, Knight, & Cross, 1997; Easterbrooks & Goldberg, 1984; Lugo‐Gill & Tamis‐LeMonda, 2008; Tamis‐LeMonda et al., 2004). Fathers who are more engaged and attentive in their interactions promote an environment for sharing social information which supports core cognitive skills. Furthermore, in the free‐play session, we did not find an association between paternal sensitivity and MDI scores. It is possible that behaviors which constitute this dimension (reciprocity, synchronicity, and positive emotion) are less likely to support cognitive skills at 24 months, given that diverse features of parenting differentially predict developmental outcomes (Roopnarine et al., 2006; Ryan et al., 2006).

During the book session, sensitive, calm, and less controlling and anxious behavior in fathers is associated with higher MDI scores in their children aged 24 months. In line with previous evidence (Cabrera, Fitzgerald et al., 2007; Cabrera, Shannon, & Tamis LeMonda, 2007; Shannon et al., 2002; Tamis‐LeMonda et al., 2004), fathers who support their children to explore and engage with objects and the world around them allow the child to acquire new information and develop their cognitive skills. On the other hand, controlling and conflictual interactions, which are linked to poor verbal input (Baumwell, Tamis‐LeMonda, & Bornstein, 1997; Radin & Epstein, 1975), may limit the child's experience of shared attention and turn‐taking, thus restricting learning behaviors in support of cognitive improvement. Our findings from the book session link to evidence which suggests that the provision of rich language experiences and educational references support cognitive and learning skills (Coley, Lewin‐Bizan, & Carrano, 2011). This requires further investigation with fathers and may support the need for targeted interventions which not only encourage father–child book reading but also provide information on ways to optimize the effects of book reading on children's cognitive development. One such model, dialogic book‐sharing (an interactive form of shared reading), significantly benefits child development (Vally, Murray, Tomlinson, & Cooper, 2015; Whitehurst et al., 1994).

Also keep in mind that data from the concurrent arm of the study were correlational and do not imply a direction of effect from parent to child. Hence, we cannot be certain whether lower MDI scores are a consequence of poorer parenting quality or whether infants with poor cognitive skills influence their father's interactions. It also is likely that an atypical social trajectory in the child would affect parents’ interactive patterns. For example, the infant's biological characteristics likely influence his or her interactive abilities and also may influence paternal behavior. Therefore, in our research design, bidirectional influences cannot be ruled out; that is, children with higher cognitive skills might elicit a more positive response in their parent, and thus increase paternal sensitivity (Bernier et al., 2010). More positive paternal interactions may in turn facilitate the child's curiosity and ability to master new skills. In contrast, a poorly regulated child or one who is less able to engage positively may fail to get the same response from his/her parent (Lunkenheimer, Kemp, & Albrecht, 2013). It also is possible that since infant and father are closely genetically related, the associations observed could be mediated through shared genetic variants, including an inherited cognitive ability and behavioral style (Tucker‐Drob, Briley, & Harden, 2013).

### Father–Child Interactions and Cognitive Development in Girls and Boys****

There was no robust evidence of a gender interaction at either study time point. This is in line with previous evidence on gender differences in other areas of child development, with some evidence to suggest that fathers treat sons and daughters similarly (Belsky, 1984; Lamb, Frodi, Hwang, & Frodi, 1982; Pougner et al., 2011). However, previous work also has indicated that sons may be more influenced by father involvement (Bronte‐Tinkew et al., 2008).

### Strengths and Limitations

The findings reported should be interpreted in light of certain limitations. First, although attrition was minimal, fathers in the study were predominantly Caucasian and middle‐class, and had relatively high levels of education. Accordingly, the generalizability of the findings to other populations may be limited somewhat. Second, although the sample was relatively large, there were a smaller number of male and female infants, and this may have limited our ability to detect gender‐related effects. Third, conclusions about the role of early father–infant interactions in children's cognitive development are constrained by the measures included in the study. Although the 2‐year coding scheme (Madden et al., 2015) was derived from an existing measure with proven reliability and validity, the measure has not received extensive psychometric evaluation. Furthermore, the reliance on a brief sequence of father–infant interactions at both study time points may limit generalizability. However, the inclusion of two different interactive settings at 24 months has highlighted different styles of paternal interactions that are linked with children's cognitive functioning.

Despite these caveats, this is the first study to examine the longitudinal association between observed father–infant interactions as early as 3 months of age and later cognitive development in children. Observational measures of father–child interactions prevented bias and measurement imprecision which would have otherwise been present in self‐report measures. Video observations were coded by trained researchers who were blinded to family characteristics; and those who coded the 2‐year interactions were blind to the 3‐month interactions, and vice versa. The MDI also was conducted by a trained researcher, allowing an accurate administration of this assessment.

In summary, the association between paternal interactions and cognitive outcome is evident at a very early age; therefore, putting preventive measures in place in early infancy to support fathers to better interact with their children is of immense importance. Moreover, fathers’ parenting is likely to mirror the parenting that they had received (Madden et al., 2015), so interventions at an individual and a policy level offer the potential to be of benefit across generations (Pougner et al., 2011).
